# The Validation of Multifactor Model of Plasma Aβ_*42*_ and Total-Tau in Combination With MoCA for Diagnosing Probable Alzheimer Disease

**DOI:** 10.3389/fnagi.2020.00212

**Published:** 2020-07-21

**Authors:** Fubin Jiao, Fang Yi, Yuanyuan Wang, Shouzi Zhang, Yanjun Guo, Wenjin Du, Ya Gao, Jingjing Ren, Haifeng Zhang, Lixin Liu, Haifeng Song, Luning Wang

**Affiliations:** ^1^Medical School of Chinese People’s Liberation Army, Beijing, China; ^2^Department of Neurology, The 2nd Medical Center, National Clinical Research Center for Geriatric Disease, Chinese People’s Liberation Army General Hospital, Beijing, China; ^3^Health Service Department of the Guard Bureau of the Joint Staff Department, Joint Staff of the Central Military Commission of Chinese PLA, Beijing, China; ^4^Department of Neurology, Lishilu Outpatient, Jingzhong Medical District, Chinese People’s Liberation Army General Hospital, Beijing, China; ^5^The Psycho Department of Beijing Geriatric Hospital, Beijing, China; ^6^Department of Neurology, Beijing Friendship Hospital, Capital Medical University, Beijing, China; ^7^Department of Neurology, Air Force Medical Center, Chinese People’s Liberation Army, Beijing, China; ^8^Department of Geriatrics, The Second Hospital of Hebei Medical University, Shijiazhuang, China; ^9^National Engineering Research Center for Protein Drugs, Beijing, China

**Keywords:** Alzheimer disease, β-amyloid, tau, Montreal Cognitive Assessment, ultrasensitive immunomagnetic reduction

## Abstract

Alzheimer disease (AD) has an insidious onset and heterogeneous clinical symptoms. The well-accepted biomarkers for clinical diagnosis of AD include β-amyloid (Aβ) deposition and pathologic tau level within cerebral spinal fluid (CSF) and imaging AD pathology such as positive emission tomography (PET) imaging of the amyloid-binding agent Pittsburgh compound B (PET-PiB). However, the high expense and invasive nature of these methods highly limit their wide usage in clinic practice. Therefore, it is imperious to develop less expensive and invasive methods, and plasma biomarkers are the premium targets. In the current study, we utilized a single-blind comparison method; all the probable AD cases met the core clinical National Institute on Aging and Alzheimer’s Association (NIA-AA) criteria and validated by PET-PiB. We used ultrasensitive immunomagnetic reduction (IMR) assays to measure plasma Aβ_*42*_ and total-tau (t-tau) levels, in combination with different variables including Aβ42 × t-tau value, Montreal Cognitive Assessment (MoCA), and Mini Mental State Examination (MMSE). We used logistic regression to analyze the effect of all these variables in the algorism. Our results showed that (1) plasma Aβ42 and t-tau are efficient biomarkers for AD diagnosis using IMR platform, whereas Aβ42 × t-tau value is more efficient for discriminating control and AD; (2) in the control group, Aβ42 level and age demonstrated strong negative correlation; Aβ42 × t-tau value and age demonstrated significant negative correlation; (3) in the AD group, t-tau level and MMSE score demonstrated strong negative correlation; (4) using the model that Aβ42, Aβ42 × t-tau, and MoCA as the variable to generate receiver operating characteristic (ROC) curve, cutoff value = 0.48, sensitivity = 0.973, specificity = 0.982, area under the curve (AUC) = 0.986, offered better categorical efficacy, sensitivity, specificity, and AUC. The multifactor model of plasma Aβ42 and t-tau in combination with MoCA can be a viable model separate health and AD subjects in clinical practice.

## Introduction

Alzheimer disease (AD) is a neurodegenerative disease with latent initiation and progressive course, which is the most common cause of dementia and the fourth cause of mortality in the elder population (Katzman, 2008). Growing evidences have shown the early diagnosis, before massive neuron loss and dementia occur, is crucial to AD treatment (Jack et al., 2013; Dubois et al., 2016). IWG 2016 and the National Institute on Aging and Alzheimer’s Association (NIA-AA) research framework are aligned on the key issue that although AD is currently defined as a biological event throughout the course, the combination of abnormal β-amyloid (Aβ) and tau biomarkers is conclusive enough to define AD regardless of cognitive symptoms (Jack et al., 2018).

Aβ level in cerebral spinal fluid (CSF) and Aβ accumulation measured by positive emission tomography (PET) imaging of the amyloid-binding agent Pittsburgh compound B (PET-PiB) have been validated as viable AD biomarkers. Absolute sensitivity of amyloid PET is comparable to the gold standard of autopsy (Roberts et al., 2017). However, the high expense and limited availability of the amyloid PET and the invasive nature of CSF collection highly limit its wide used in clinic practice (Villemagne et al., 2013; Vos et al., 2013; Sperling et al., 2014; Baird et al., 2015). Therefore, it is imperious to develop less expensive and invasive methods, and plasma biomarkers are the premium targets. Moreover, blood biomarkers such as plasma total tau (t-tau) have shown promising potential to identify neurodegenerations, whereas plasma Aβ42 was shown to be a high-performance biomarker for AD (Dage et al., 2016; Ovod et al., 2017; Nakamura et al., 2018). However, the sensitivity and specificity of blood biomarkers detection will determine the future of their clinical application, thus needing further clinical validation.

Currently, many technologies have been developed for detecting plasma Aβ42, t-tau: ELISA, CLIA, ultrasensitive immunomagnetic reduction (IMR), and so on. Immunomagnetic reduction has shown far better sensitivity than others, with clinical validation (Chiu et al., 2012, 2013; Chen et al., 2019; Lue et al., 2019a). But more clinical validation is needed to determine the reliability of IMR in AD diagnosis.

In our study, 97 cases of volunteers were discretely screened and diagnosed by experienced neurologists, to guarantee the quality for selected subjects. Through cognitive psychophysiology evaluation analysis and confirmed by PET-PiB neuroimaging analysis, all the AD patients met the core clinical NIA-AA criteria. We used IMR technology measuring plasma Aβ42 and t-tau, in combination with other factors, and thus comprehensively evaluated the sensitivity and specificity of AD diagnosis, which paved the avenue of potential using IMR technology for AD early diagnosis.

## Materials and Methods

### Participants

Alzheimer disease group: 40 AD patients were recruited from the neurology department of PLA hospital between August 2017 and June 2018. All the patients underwent cognitive psychology scale evaluation and neuroimaging measurement and confirmed by PET-PiB examination. All probable AD patients met the core clinical NIA-AA criteria (Jack et al., 2011), without family history.

Normal control (NC) group: 57 healthy volunteers were recruited. All the subjects have normal cognitive function by neuropsychology evaluation, also without severe heart, liver, kidney, or other systematic diseases; mental illness; surgery; and injury history or other major disease history, without dementia patients in the family.

Consensus agreements were signed by patients himself/herself or custodians and provided blood samples; the entire process met human rights, humanity, and medical ethical standard.

### Methods

Sample collection: 5 mL non-fasting venous blood sample (K2 EDTA tube) was drawn from every subject. The blood samples were centrifuged at 2,500 × *g* for 15 min within 3 h of collection, and plasmas were aliquoted into cryotubes (1 mL per tube) and stored at −80°C. Each sample was assigned an identification number following reception. The laboratory staffs handling the sample processing were blind to the clinical status and the demographic data of the subjects.

The Mini Mental State Examination (MMSE) (Li et al., 2016) and Montreal Cognitive Assessment (MoCA) Beijing version^1^ (Lu et al., 2011) evaluation were performed by the same doctor for the entire study: in the normal age range (score > 1.5 standard deviation), age- and education-matched normal recipient, and CDR = 0. Every patient underwent magnetic resonance imaging (MRI) and PET-PiB imaging scan examination. Aβ42 and t-tau concentration were measured using IMR technology for all the collected plasma samples.

Reagents: Calibrator60 (Aβ42 standard), MagQu, CA-DEX-0080; tau IMR Reagent, MagQu, MF-tau-0060; tau Solution-L (standard L), MagQu, CL-tau-000T; tau Solution-H (standard H), MagQu, CL-tau-050T; Aβ42 IMR Reagent, MagQu, MF-AB2-0060; Aβ42 Solution-M (standard M), MagQu, CL-AB2-020T; 6 × 50 mm sample analysis tubes, MagQu, MQ-TUB-0100; disposable vacuum blood collection tube (K2 EDTA tube), Jiangsu Yuli Medical Instrument Co. Ltd., Y30983502; Cryo tube, CORNING, 430659.

Immunomagnetic reduction measurements: Details of the mechanism and technology of IMR have been previously reported (Nasreddine et al., 2005; Lee et al., 2016; Yang et al., 2017b). The reagents used to determine plasma Aβ and t-tau protein levels in this study consisted of dextran-coated Fe3O4 nanoparticles functionalized with antibodies. The percentage reduction in an alternating current (ac) that reflects the magnetic susceptibility (Xac) of a reagent due to the interactions of functionalized magnetic nanoparticles and target proteins. The percentage reductions of immunomagnetic signals are then converted to target protein concentrations using the standard curves of the respective analyses. The selection of the antibodies conjugated to the IMR reagents was based on epitopes, affinity to antigens, ability to be conjugated onto nanoparticles, and the ability to provide linearity of standard curves quantified by magnetic signal reduction. For t-tau assay, 40 μL of plasma sample was mixed with 80 μL IMR reagents at room temperature, and for Aβ42 assay, 60 μL of plasma sample was mixed with 60 μL of IMR reagent.

### Statistical Analysis

Data were expressed as means ± SD or an absolute number with a proportion for descriptive statistics. Spearman correlation analysis was applied to examine the correlations between plasma Aβ measures and cerebral uptake values of ^11^C Pittsburgh Compound B PET, ^11^C-PiB PET in different brain regions. Significant correlations were validated using non-parametric Spearman rank-order correlations. Correlation analysis was also used to evaluate the correlations between the plasma Aβ measures, ^11^C-PiB PET SUVRs and each parameter of the demographic data, clinical characteristics, and cognitive tests. Multiple linear regression analysis was used to further evaluate the associations between plasma Aβ measures and ^11^C-PiB PET binding after controlling for age. A *P*-value of 0.05 was defined as the threshold of statistical significance in each test. To elucidate the AD-related tests, the current study used statistical analysis with SPSS for data analysis; considering the data do not obey Gaussian distribution, we used Spearman correlation analysis, to describe the level and direction of the correlation of two variables (*r* > 0 represents positive correlation, *r* < 0 represents negative correlation, the closer *r* value to 1, the stronger correlation is, and *P* < 0.05 represents a significant difference). Within the final prediction model, binary logistic regression analysis demonstrated the result of (*P*_MoCA_, *P*_Aβ 42_, and *P*_Aβ 42 × t–tau_) < 0.05, whereas (*P*_MMSE_ and *P*_t–tau_) > 0.05. Considering the stability of using this model, we decided to choose Aβ42, Aβ42 × t-tau and MoCA these three variables as prediction factors.

## Results

### Patients Selection

Ninety-seven volunteers aged between 54 and 78 years (mean = 68.0 ± 9.3) were recruited, of which 57.7% were females. The control group had 57 subjects; the AD group had 40 subjects, with an average age of 67.9 ± 9.5 and 68.1 ± 9.0 years, respectively. Average MMSE value was 28.25 ± 3.36 in control group and 12.67 ± 9.21 in AD group; Average MoCA value was 26.25 ± 4.61 in control group and 10.69 ± 7.33 in AD group, therefore, both MMSE (*P* = 1.41 × 10^–19^) and MoCA (*P* = 8.81 × 10^–22^) showed highly significant difference between control and AD group (Table 1).

**TABLE 1:** Statistic detail of the recruits.

**Group**	**NC (57)**	**AD (40)**	***P***	**Overall (97)**
Male: female	26:31	15:25	0.53	41:56
Age, years	67.9 ± 9.5	68.1 ± 9.0	0.67	68.0 ± 9.3
MMSE	28.25 ± 3.36	12.67 ± 9.21	1.41 × 10^–19^	21.78 ± 10.19
MoCA	26.25 ± 4.61	10.69 ± 7.33	8.81 × 10^–22^	19.79 ± 9.84
Plasma t-tau, pg/mL	20.65 ± 3.52	25.91 ± 8.12	3.42 × 10^–5^	22.82 ± 6.01
Plasma Aβ42, pg/mL	16.92 ± 1.67	18.77 ± 1.93	2.31 × 10^–6^	17.68 ± 1.99
Aβ42 × t-tau	352.53 ± 90.88	490.44 ± 190.48	7.03 × 10^–6^	409.40 ± 152.38
Aβ42/ t-tau	0.83 ± 0.11	0.77 ± 0.18	0.04	0.81 ± 0.14

*Clinical characters, mean ± SD. AD, Alzheimer disease; NC, normal control; MMSE, Mini-Mental State Examination; MoCA, Montreal Cognitive Assessment.*

### IMR Measurement of Plasma Aβ42 and T-tau Levels

**FIGURE 1:**
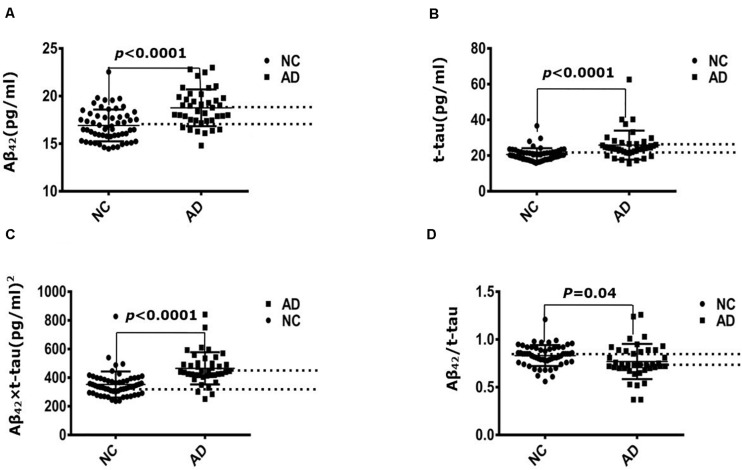
Immunomagnetic reduction measurement of plasma Aβ42 and t-tau protein levels. **(A)** Aβ42 concentration of NC and AD group; **(B)** t-tau concentration of NC and AD group; **(C)** Aβ42 × t-tau value of NC and AD group; **(D)** Aβ42/t-tau value of NC and AD group. NC: normal control (*n* = 57); AD, Alzheimer disease (*n* = 40).

Immunomagnetic reduction technology was used to measure the human plasma Aβ42 and t-tau levels. Table 1 demonstrated that the mean value of t-tau concentration was 20.65 ± 3.52 pg/mL in the control group and 25.9 ± 8.12 pg/mL in the AD group; the difference was strongly significant (*P* = 3.42 × 10^–5^). Mean value of Aβ42 concentration was 16.92 ± 1.67 pg/mL in the control group and 18.77 ± 1.93 pg/mL in the AD group; the difference was strongly significant between control and AD group (*P* = 2.31 × 10^–6^). Mean value of Aβ42 × t-tau was 352.53 ± 90.88 pg/mL in the control group and 490.44 ± 190.48 pg/mL in the AD group; the difference was strongly significant (*P* = 7.03 × 10^–6^). Mean value of Aβ42/t-tau concentration was 0.83 ± 0.11 in the control group and 0.77 ± 0.18 in the AD group; the difference was strongly significant (*P* = 0.04). Figure 1 demonstrates that all the differences between two groups were strongly significant.

### Receiver Operating Characteristic Curve Analysis

**FIGURE 2:**
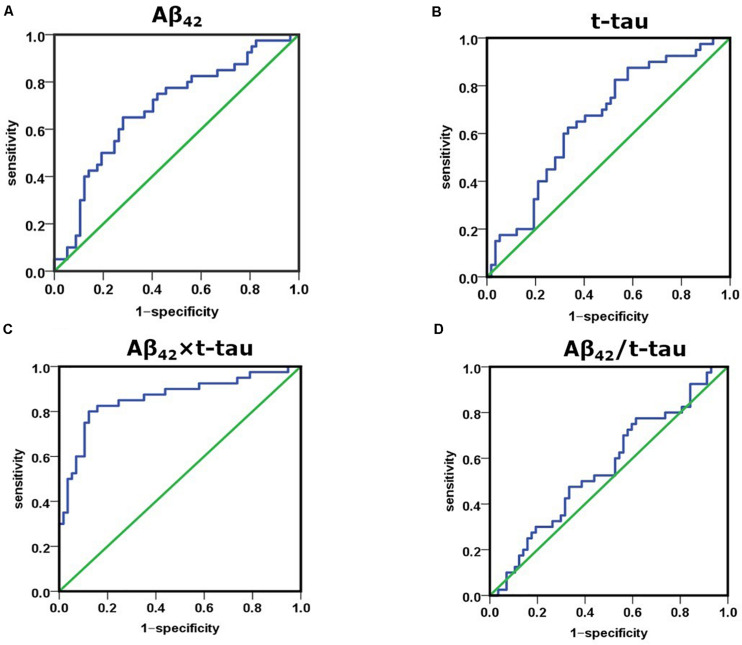
Receiver operating characteristic curve analysis for discriminating between NC and AD subjects using Aβ42 level, t-tau level, Aβ42 × t-tau value, and Aβ42/t-tau value as diagnostic parameters. **(A)** AUC of Aβ42 concentration between NC and AD group; **(B)** AUC of t-tau concentration between NC and AD group; **(C)** AUC of Aβ42 × t-tau value between NC and AD group; **(D)** AUC of Aβ42/t-tau value between NC and AD group. NC, normal control (*n* = 57); AD, Alzheimer disease (*n* = 40).

Receiver operating characteristic (ROC) curve analysis demonstrated that the cutoff value of Aβ42 concentration between control and AD group was 17.22 pg/mL, sensitivity was 0.650, specificity was 0.719, and area under the curve (AUC) was 0.689 (Figure 2A). The cutoff value of t-tau concentration between control and AD group was 21.30 pg/mL, sensitivity was 0.625, specificity was 0.667, and AUC was 0.659 (Figure 2B). The cutoff value of Aβ42 × t-tau between control and AD group was 403.72 (pg/mL)^2^, sensitivity was 0.825, specificity was 0.842, and AUC was 0.883 (Figure 2C); The cutoff value of Aβ42/t-tau between control and AD group was 0.74, sensitivity was 0.775, specificity was 0.386, and AUC was 0.558 (Figure 2D). The values were summarized in Table 2. A biomarker combination of Aβ42 × t-tau has the highest sensitivity, specificity, and AUC.

**TABLE 2:** ROC curve analysis of AD and control subjects.

	**Cutoff**	**Sensitivity**	**Specificity**	**AUC**
Aβ42	17.22	0.650	0.719	0.689
t-tau	21.30	0.625	0.667	0.659
Aβ42 × t-tau	403.72	0.825	0.842	0.883
Aβ42/t-tau	0.74	0.775	0.386	0.558

*AD, Alzheimer disease; NC, normal control; AUC, area under the receiver operating characteristic curve.*

### Spearman Correlation Analysis

Spearman correlation analysis in the control group showed that Aβ42 concentration and age demonstrated a strong negative correlation (*r* = −0.365, *P* = 0.005); Aβ42 × t-tau value and age demonstrated significant negative correlation (*r* = −0.266, *P* = 0.046) (Table 3). In the AD group, t-tau concentration and MMSE score demonstrated a strong negative correlation (*r* = −0.579, *P* = 0.006) (Table 4). This thus suggests that in AD patients, the lower MMSE value is, which represents the severity of the disease, the higher is the plasma t-tau protein level.

**TABLE 3:** Correlation analysis of NC group.

	**MMSE**		**MoCA**		**Age**	
	***r***	***p***	***r***	***p***	***r***	***p***
Aβ42	−0.085	0.530	0.020	0.880	−0.365**	0.005
t-tau	−0.151	0.263	−0.081	0.550	−0.105	0.437
Aβ42 × t-tau	−0.157	0.244	−0.047	0.731	−0.266*	0.046

*NC, normal control; MMSE, Mini Mental State Examination; MoCA, Montreal Cognitive Assessment.*

**TABLE 4:** Correlation analysis for MMSE, MoCA < 10 of AD group.

	**MMSE**		**MoCA**		**Age**	
	
	***r***	***P***	***r***	***p***	***r***	***p***
Aβ42	0.070	0.762	0.109	0.638	0.274	0.229
t-tau	−0.579**	0.006	−0.336	0.137	−0.016	0.944
Aβ42 × t-tau	−0.187	0.416	−0.197	0.391	−0.046	0.844

*AD, Alzheimer disease; MMSE, Mini Mental State Examination; MoCA, Montreal Cognitive Assessment.*

### Logistic Regression Analysis

**FIGURE 3:**
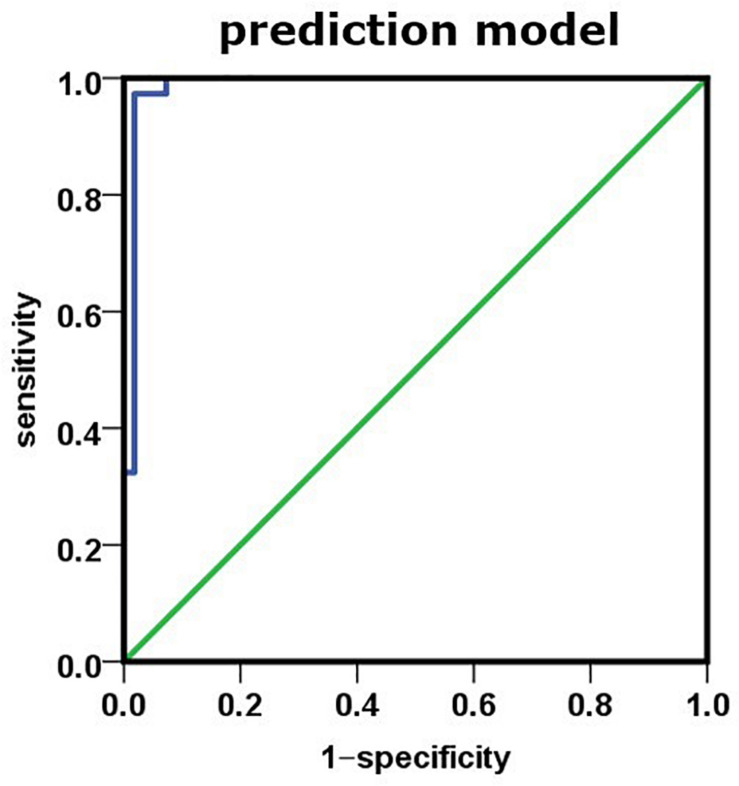
Using prediction value of the model as variable, PET-PiB as standard; the ROC curve is as shown.

To further study the relationship between plasma biomarker and brain Aβ deposit, we utilized SPSS software performing logistic regression analysis. We combined different variables to establish a disease prediction model (i.e., practical clinical diagnosis) and predicted if the individual is AD or NC who met the entry criteria (i.e., clinical diagnosis). Our model introduced the variables Aβ42, t-tau, Aβ42 × t-tau, MMSE, and MoCA predicted as shown in Table 5. This model has the prediction accuracy of 98.2% for the control groups and 97.3% for the AD group, an overall prediction percentage of 97.8%. Further variable logistic analysis concluded that Aβ42, Aβ42 × t-tau, and MoCA also had statistical significance in the model (*P* < 0.05), as the prediction accuracy of these three variables shown in Table 6. This three-variable model has a prediction accuracy of 98.2% for the control groups and 94.6% for the AD group, an overall prediction percentage of 96.7%. Using the prediction values of Aβ42, Aβ42 × t-tau, and MoCA as variable, with PET-PiB as standard, the ROC curve is shown in Figure 3, as cutoff value = 0.48, sensitivity = 0.973, specificity = 0.982, AUC = 0.986. The results demonstrated the prediction model of Aβ42, Aβ42 × t-tau, and MoCA have a significant correlation with clinical probable AD diagnosis.

**TABLE 5:** Binary logistic regression analysis for MoCA, MMSE, Aβ42, t-tau, and Aβ42 × t-tau variables.

**Actual detection**		**Forecast**		
		**Clinical diagnosis**		**Accuracy rate**
		**0**	**1**	
Practical clinical diagnosis	0	54	1	98.2
	1	1	36	97.3
Overall accuracy				97.8

*0: normal control; 1: Alzheimer disease.*

**TABLE 6:** Binary logistic regression analysis for Aβ42, Aβ42 × t-tau, and MoCA variables.

**Actual detection**		**Forecast**		
		**Clinical diagnosis**		**Accuracy rate**
		**0**	**1**	
Practical clinical diagnosis	0	54	1	98.2
	1	2	35	94.6
Overall accuracy				96.7

*0: normal control; 1: Alzheimer disease.*

## Discussion

As all we know, the early diagnosis and treatment of AD may benefit to improve symptoms and decelerate the course and also alleviate the burden of the family and society (Andreasen et al., 2001; Kim et al., 2010). Therefore, it is crucial to define the disease with a clear biological foundation including clinical and preclinical phases. The NIA-AA Research Framework focuses on biomarker discovery in living people for AD diagnosis, including biomarkers for Aβ deposition, pathologic tau, neurodegeneration, and so on (Jack et al., 2018). CSF AD-specific pathological protein test and PET have become internationally recognized biomarker diagnoses in living patients (Prestia et al., 2013; Toledo et al., 2014; Vos et al., 2015). Especially, the absolute sensitivity of amyloid PET relative to an autopsy gold standard has been assessed (Roberts et al., 2017); its sensitivity and accuracy are higher than neuropsychological evaluation, computed tomography and MRI. However, CSF biomarker analysis related lumbar puncture is an invasive procedure and poor in repeatability, and the assessment is not always feasible and difficult to be accepted by patients in some countries, especially in the elderly patients. Meanwhile, brain imaging biomarkers (amyloid-PET, tau-PET) also have their disadvantages: the assay cost is too expensive, and it requires sophisticated technologies and equipment, which are not accessible in regular clinical settings, which limit the widespread application of these two types of biomarkers. Therefore, compared with all above, analyzing pathologic Aβ and tau in peripheral blood, which have great advantages, including blood samples being easy to access, the procedure is non-invasive or minimally invasive, with reasonable cost-effective and good replication rate. More and more studies show that blood biomarkers have promising prospects (Kaneko et al., 2014; Mattsson et al., 2016; Nabers et al., 2018; Nakamura et al., 2018).

Immunomagnetic reduction technology for AD-associated plasma biomarkers has been developed in the last few years (Lue et al., 2019a). In a recent study, plasma Aβ42 level was assayed by IMR in European Caucasian AD subjects (Teunissen et al., 2018). However, pathologic Aβ and t-tau levels are under gene regulation; there are interracial discrepancies of these gene expressions (Huynh and Mohan, 2017). Thus, directly using the data from the European population in the Chinese population will have great limitations. Currently, there are many studies assessed the potential of Aβ40, Aβ42, and t-tau measured by IMR in discriminating AD in China Taiwan area (Chiu et al., 2012, 2013; Yang et al., 2017a; Fan et al., 2018). These studies used the same methods but obtained different cutoff values, which may be caused by race, regions, living habit differences, and so on. Therefore, a larger sample size from mainland China is needed for establishing accurate cutoff value for the Chinese population. In this study, we used IMR technology to measure plasma Aβ42 and t-tau protein levels and for the first time verified the reliability of the multiparameter model of plasma Aβ42 and t-tau in combination with MoCA for diagnosing probable AD confirmed by PET-PiB, which provides sensitive and reliable discrimination between control and AD subjects in a Chinese population.

In this single-blind test, we discovered plasma Aβ42, t-tau, and Aβ42 × t-tau levels are significantly higher in AD subjects compared to the control subjects. Further ROC curve analysis demonstrated that plasma Aβ42 × t-tau as a combinational biomarker yielded better discrimination between control and AD subjects than that of plasma Aβ42 or t-tau concentration alone. Using Spearman correlation analysis, we found Aβ42 level is negatively correlated with age (*r* = −0.365, *P* = 0.005), and the value of Aβ42 × t-tau is negatively correlated with age (*r* = −0.266, *P* = 0.046) in the control group. These findings suggest that Aβ42 level as well as Aβ42 × t-tau value decreases according to age growth in healthy subjects, which agree with those of Lue et al. (2019b), who showed that plasma Aβ42 decreased as age increased in cognitively normal subjects. These relationships provided evidence that Aβ42 may be involved in the pathophysiology of aging and could act as plasma biomarkers in aging-associated disease. In our study, in AD patients whose MMSE/MoCA value is less than 10, the t-tau level is strongly negatively correlated with MMSE value (*r* = −0.579, *P* = 0.006). Our findings suggest that, in AD patients, the lower the MMSE value is, the higher plasma the t-tau protein level is, which is consistent with the discovery from clinical studies that showed MMSE score is negatively correlated with plasma t-tau concentration (Mattsson et al., 2016). That reveals the severity of AD is positively correlated with the level of t-tau.

To establish the valid prediction model, we initially started with a five-variable model, including Aβ42, t-tau, Aβ42 × t-tau, MMSE, and MoCA. Our prediction model can distinguish AD subjects from control subjects with an accuracy of 98.2 and 97.3%, respectively, and the overall prediction rate is 97.8%. To further simplify our model, we tried different combinations, and discovered three variables, Aβ42, Aβ42 × t-tau, and MoCA, are sensitive enough to yield similar prediction accuracy in comparison to a five-variable prediction model (98.2% for the control group, 94.6% for AD group and the overall 96.7%). Moreover, MoCA is a superior screen tool for the identification of mild cognitive impairment compared to MMSE (Li et al., 2018; Pinto et al., 2019). Therefore, the three-variable model is optimal for predicting early stage AD with MoCA as its neuropsychological measurement. Further ROC curve analysis showed using a three-variable model offered better categorical efficacy, sensitivity, specificity, and AUC compared with using Aβ42 × t-tau alone. We think that the prediction model established by plasma biomarkers has a significant correlation with clinical diagnostics. Lue et al. (2017), using the IMR assay, found that the regression model incorporating age and tau level has 81 and 96% accuracy for identifying probable AD in Banner Sun Health Institute (BSHRI) and National Taiwan University Hospital (NTUH) cohorts, respectively; incorporating age with the products of Aβ42 and tau in BSHRI and NTUH cohorts have 84 and 95% accuracy, respectively. At a 382.68 (pg/mL)^2^ cutoff value, the product of Aβ42 and tau achieved 92% accuracy with 96% sensitivity and 90% specificity in identifying AD in the combined cohorts. Compared to this study, we relied on PET-PiB, a gold standard for AD in living patients, as one of the criteria for the recruitment of AD patients, and created a new prediction model of AD. This model obtained different cutoff values from the United States and China Taiwan area, and the sensitivity and specificity are more than 95%. The strength of the study is the predictive value of the combination of different parameters. In AD, Aβ and tau proteins are the core biomarkers. Significant increases in Aβ42 and t-tau levels in plasma have been found in early AD patients (Chen et al., 2019; Lue et al., 2019a). Our attempt of combining these two pathological proteins with neuropsychological measurement offers a new avenue of enhancing sensitivity and specificity for diagnosing with plasma biomarkers only, thus overcoming the limitation of plasma biomarkers, as described by Chen et al. (2019) for plasma Aβ42 and t-tau correlate with cognitive decline in amnestic MCI patients.

Although the current study has plausible results, it does have certain limitations. First, the volunteer number included in the study is rather small, thus requiring to expand sample size for further validation of our data and model. Second, the current study is a retrospective study; a further prospective longitudinal study will be extremely valuable.

In conclusion, our study introduced variables Aβ42, t-tau, Aβ42 × t-tau, MMSE, and MoCA into the model and performed analysis; analyzed the role of each variable in the model via logistic regression; further determined using Aβ42, Aβ42 × t-tau, and MoCA for companion diagnosis for AD; compared with previous using a single biomarker; and greatly enhanced the efficacy and stability of the diagnosis. Moreover, as blood samples are more accessible than CSF in community hospitals, plasma biomarkers for early diagnosis of AD can be promoted and popularized. Although early screening and diagnosis of AD are unlikely to rely on a single biomarker or a combination of biomarkers in blood, this information may serve a crucial role for companion diagnosis, so as to give early warning and early intervention for AD for clinicians.

## Data Availability Statement

All datasets generated for this study are included in the article/supplementary material.

## Ethics Statement

The studies involving human participants were reviewed and approved by the Medical Ethics Committee of Chinese People’s Liberation Army General Hospital. The patients/participants provided their written informed consent to participate in this study.

## Author Contributions

LW and HS designed the study strategy. YW, SZ, YG, WD, HZ, and LL recruited the participants and collected their information and blood samples. FJ, FY, YW, and JR performed the experiments. FJ, FY, and YG performed the data analysis, data management, and reference collection. FJ, FY, and JR wrote the manuscript. All authors reviewed the manuscript.

## Conflict of Interest

The authors declare that the research was conducted in the absence of any commercial or financial relationships that could be construed as a potential conflict of interest.

## Footnotes

1 www.mocatest.org
